# Lack of Correlation Between Liver Tests Abnormalities and Trabectedin Efficacy in the Treatment of Soft Tissue Sarcoma: a Retrospective Study

**DOI:** 10.1038/srep12077

**Published:** 2015-08-03

**Authors:** Bruno Vincenzi, Luciano Stumbo, Giuseppina Maltese, Linda Cerbone, Mariella Spalato Ceruso, Giuseppe Badalamenti, Daniele Santini, Giuseppe Tonini, Anna Maria Frezza, Delia De Lisi, Marianna Silletta

**Affiliations:** 1 Medical Oncology, University campus Bio-Medico, Rome, Italy; 2 Department of Medical Oncology, Policlinico P. Giaccone, Palermo, Italy; 3 Medical Oncology, Ospedale San Camillo Forlanini, Rome, Italy

## Abstract

Elevation in liver transaminases is common in patients treated with the marine antitumor agent trabectedin. However, the impact of trabectedin-related transaminase elevations on treatment outcomes is unclear. This retrospective study investigated the correlation between liver tests abnormalities and treatment outcomes in patients with unresectable advanced or metastatic soft tissue sarcomas (STS) treated with trabectedin 1.5 mg/m^2^ once every 3 weeks at three reference centers in Italy. The effect of grade 3/4 elevations in alanine aminotransferase (ALT) or aspartate aminotransferase (AST) during the first two cycles and at any time during trabectedin treatment on progression-free survival (PFS) and overall survival (OS) were analyzed. Liver tests abnormalities during the first two cycles of chemotherapy or at any time during trabectedin treatment did not significantly affect PFS or OS. Nor were survival outcomes significantly different in the subgroups of patients with or without ALT/AST increases or with ALT/AST elevations ≥15 × the upper limit of normal (ULN) versus those with ALT/AST elevation <15 × ULN. Although liver tests abnormalities are common in patients treated with trabectedin, elevations in ALT and AST are usually transient, occur during the first two cycles of treatment, and do not appear to affect survival.

Soft tissue sarcomas (STS) are a rare group of tumors, arising predominantly from mesenchymal tissue with heterogeneous differentiation. They account for approximately 1% of all malignancies. Prognosis in advanced inoperable STS is poor, with a median survival ranging from 11 to 18 months from diagnosis of advanced disease^1^. Current treatment guidelines recommend anthracycline-based chemotherapy, primarily doxorubicin alone or in combination with ifosfamide, as the first-line treatment for advanced STS^2^. Therapeutic options after failure of doxorubicin and/or ifosfamide are limited.

Trabectedin (Yondelis®, PharmaMar, Spain), a cytotoxic compound isolated from the marine tunicate *Ecteinascidia turbinata*, is an active drug for the treatment of patients with locally advanced or metastatic STS after failure of standard therapy^3^,^4^,^5^,^6^. Trabectedin, at the dose of 1.5 mg/m^2^ once every 3 weeks given as a 24-hour infusion, was approved in 2007 by the European Medicines Agency (EMA) for the treatment of adult patients with advanced STS, after failure of anthracyclines and ifosfamide, or who are unsuited to receive these agents^7^. Liver tests abnormalities (i.e. elevation in liver transaminases) affecting up to 40% of patients are among the most common adverse events (AEs) associated with trabectedin^8^,^9^,^10^,^11^. Trabectedin commonly induces transient, self-limiting aspartate aminotransferase (AST)/alanine aminotransferase (ALT) elevations, that are easily manageable and non-cumulative^10^,^12^,^13^.

The objective of this study was to investigate the correlation between liver tests abnormalities and trabectedin efficacy in the treatment of patients with STS, by evaluating the impact of trabectedin-related liver adverse events on treatment outcomes.

## Materials and Methods

### Study design

This was an observational retrospective analysis of the data from patients with STS in treatment with trabectedin 1.5 mg/m^2^ once every 3 weeks, as a 24-hour continuous intravenous (i.v.) infusion, in three reference centers in Italy.

### Patients

All patients enrolled in the study were ≥18 year-olds and were required to have unresectable advanced or metastatic, histologically proven STS measurable in accordance with the Response Evaluation Criteria in Solid Tumours (RECIST v.1.1^14^). Other eligibility criteria included an Eastern Cooperative Oncology Group (ECOG) performance status (PS) score 0–2; adequate cardiac function, and adequate hematological (hemoglobin ≥9 g/dl; absolute neutrophil count ≥1.5 × 10^9^/L; platelets ≥100 × 10^9^/L), renal (serum creatinine ≤1.5 mg/dL) and hepatic function [bilirubin ≤ upper limit of normal (ULN); AST/ALT ≤2.5 × ULN; alkaline phosphatase (AP) ≤2.5 × ULN (if total AP >2.5 × ULN, AP liver fraction and/or gamma glutamyltransferase and/or 5′-nucleotidase had to be ≤ULN) and albumin >25 g/L].

Patients were excluded if they had any malignancy within the previous five years (except for basal cell carcinoma or treated cervical carcinoma *in situ*), or other relevant clinical conditions (active infection, active viral hepatitis or chronic liver disease, congestive heart failure or angina pectoris, myocardial infarction within the previous year, uncontrolled arterial hypertension, arrhythmias or abnormal left ventricular ejection fraction). Pregnant or breast-feeding women or patients not using appropriate contraceptive measures were excluded.

All patients were given standard steroid premedication to improve treatment tolerability, with dexamethasone 4 mg orally twice daily on days –1, +1 and +2. In addition, an i.v. bolus of 20 mg of dexamethasone was also administered approximately 20 minutes before the start of trabectedin infusion.

The study was conducted with the approval of Campus Bio-Medico of Rome ethical committee and signed informed consents were obtained from all study participants before registration. The methods were carried out in accordance with the approved guidelines.

Safety was evaluated in all patients receiving at least one dose of trabectedin, by assessment of adverse events (AEs), laboratory test results, physical examinations and vital signs. Laboratory values and AEs were graded according to the National Cancer Institute-Common Toxicity Criteria for Adverse Events, v.3.0.

### Study assessments

Liver toxicity was defined as grade 3/4 elevations in alanine aminotransferase (ALT) or aspartate aminotransferase (AST) [grade 3 elevation: >5–20 × ULN; grade 4 elevation: >20 × ULN]^15^.

The impact of liver tests abnormalities on treatment outcomes during treatment was analyzed by comparing time-to-event endpoints [e.g., progression-free survival (PFS) and overall survival (OS)] of patients with and without grade 3/4 ALT or grade 3/4 AST elevations during the first two cycles of treatment with trabectedin.

The analysis also compared the survival of patients with grade 3/4 ALT and/or AST elevations occurring at any time during treatment versus patients without such elevations, and of patients with peak ALT and/or AST elevations ≥15 × ULN versus patients with peak elevations <15 × ULN.

Data were collected using a standard database (Microsoft Excel Software).

### Statistical analysis

Correlation between patient characteristics and AST/ALT elevation was evaluated using two tailed Chi square test. Tumor response was assessed every two cycles according to RESCIST criteria. The disease control rate (DCR) was defined as the percentage of patients with an objective response and/or stable disease (SD) lasting ≥6 months. Progression free survival (PFS) was calculated as the period from the date of starting treatment to the first observation of disease progression or to death from any cause. The response duration was defined as the period of time from the initiation of treatment (in a patient responding to therapy) until documentation of radiological or symptomatic disease progression. The overall survival (OS) time was calculated as the period from the date of starting treatment until death from any cause or until the date of the last follow-up, at which point data were censored. PFS and OS curves were estimated by using the Kaplan-Meier method.

Stratified permutation tests were carried out to explore the association between tumor response and the presence of liver tests abnormalities.

The cut-off point for survival data was May 2014. SPSS software (version 17.00, SPSS, Chicago, USA) was used for statistical analysis. A *P* value of less than 0.05 was considered to indicate statistical significance.

## Results

### Patient population

Data were retrospectively collected by three Italian reference centers and represented the whole number of cases treated with trabectedin with enough data on liver enzyme alteration (i.e., AST, ALT, Total and fractionated Bilirubin, Alkaline Phosphatase and GGT evaluated at least once during the interval between two consecutive and always before each following course) and sufficient data about follow-up and outcome (i.e., available data about radiological response and at least 6 months of follow-up). The analysis included a total of 113 patients, enrolled between January 2010 and June 2014, with metastatic or locally-advanced inoperable anthracycline-pretreated STS. In this timeframe 12 additional patients were originally included in the database but, before the final analysis, they were excluded for incomplete data about follow-up (n = 8) and insufficient data about liver toxicity (as previously defined).

A total of 62.8% (*n* = 71) and 37.2% (*n* = 42) of patients were male and female, respectively, with a median age of 57 (range: 27–79) years and with different histological subtypes of STS. The demographic and clinical characteristics of the study population are summarized in Table 1. A median of 4 cycles of trabectedin per patient were administered (range 1–32). Fifty-three patients received 6 or more cycles of trabectedin.

### Liver function test abnormalities

No patients showed AST/ALT grade 1 elevation before receiving trabectedin. After two cycles of treatment with trabectedin, grade 3/4 elevations of ALT and/or AST were reported in 45 of 113 patients (39.82%). Grade 3 ALT and/or AST increase was observed in 33 patients and grade 4 in 12. In the 45 patients who developed liver tests abnormalities, the median ALT increase was 18 × ULN (95% CI: 8–22), and the median increase of AST was 14 × UPL (95% CI: 4–19). 39 patients had an increase of ALT and/or AST levels of more than 15 × ULN. No differences in terms of incidence of ALT/AST increase were detected according to basal patients features (age, sex, ECOG PS, number of previous chemotherapy lines, sarcoma histology).

In addition we evaluated the correlation between the presence of liver metastases and the development of liver alteration tests and no difference was detected between patients with liver metastases and patient without.

### Impact of elevated liver enzymes on response

Overall response rate (ORR) in the whole patient population was 16.8% (19 patients out of 113). However, a higher DCR was identified in 55 patients (48.7%). Therefore we explored the response rate according to the development of liver tests abnormalities (defined as ALT and/or AST elevation as previously described in the materials and methods section). In the cohort of patients with grade 3/4 ALT/AST increase (45 patients) the ORR was not statistically different than that observed in the remaining population (17.5% vs. 16.2%, *p* = 0.973). Similarly, no statistically significant difference was detected between patients who developed an increase of >15 × ULN of ALT/AST circulating levels (ORR: 15.4% vs. 17.6%, *p* = 0.976).

Even in terms of DCR, no differences were detected in both the groups of patients with grade 3/4 ALT/AST increase and those with >15 × ALT/AST increase, in comparison with the remaining population. In patients with grade 3/4 increase DCR was identified in 20 patients (20/45 patients; 44.4%), while in the cohort without grade 3/4 ALT/AST increase, DCR was detected in 55 patients (51.5%) with a *P* value of 0.590. Finally, considering the population of patients with ≥15 × ALT/AST, DCR was identified in 18 (18/39 patients; 46.2%) cases vs. 37 (50%) in the remaining population (*p* = 0.849).

### Impact of elevated liver enzymes on survival

**Figure 1:**
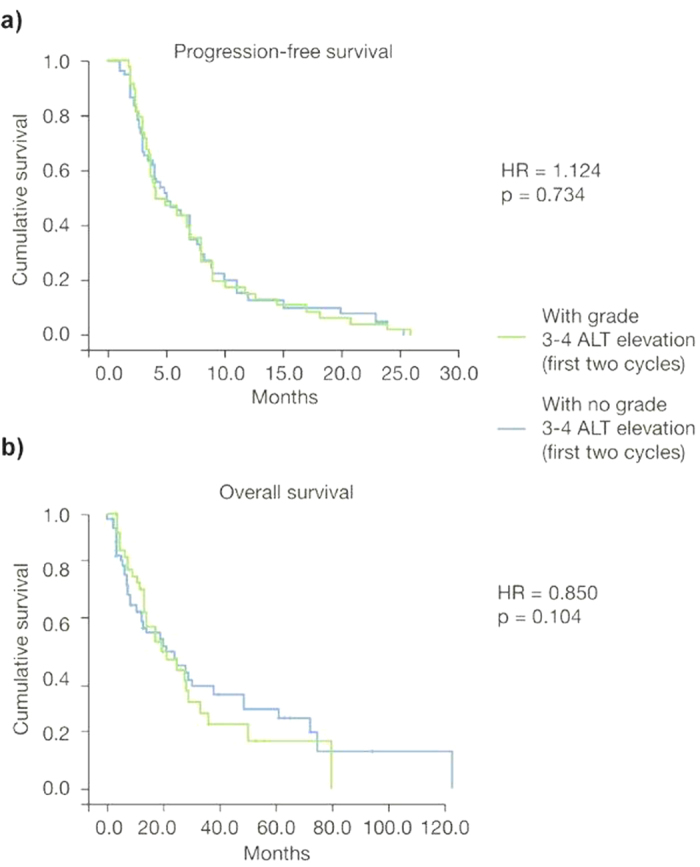
Kaplan-Meier plots of progression-free survival (**a)** and overall survival (**b)** in patients with grade 3/4 alanine aminotransferase (ALT) elevation during the first two cycles of trabectedin (*n* = 45) versus patients with no grade 3/4 ALT elevation (*n* = 68).

**Figure 2:**
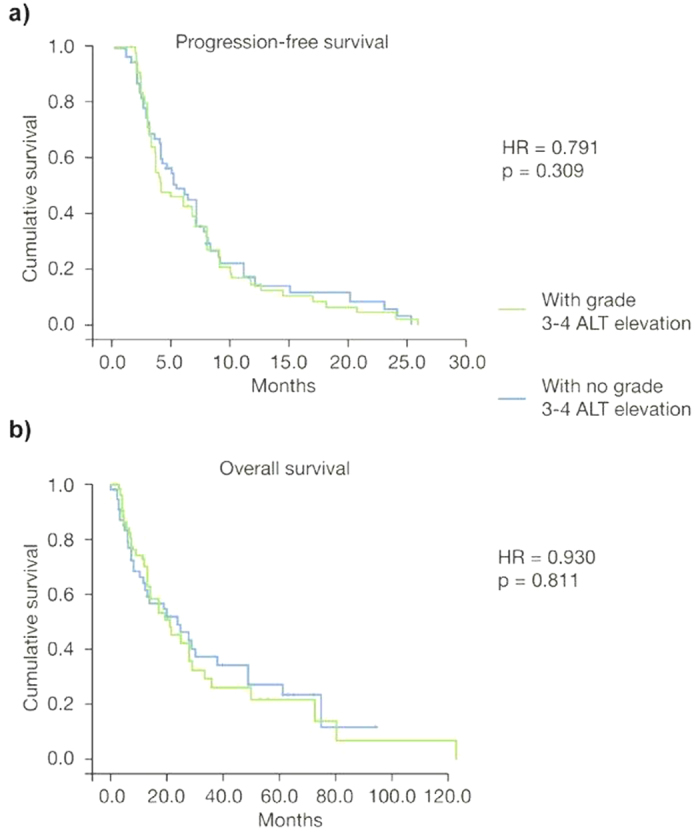
Kaplan-Meier plots of progression-free survival (**a)** and overall survival (**b)** in patients with grade 3/4 alanine aminotransferase (ALT) elevation during treatment with trabectedin (*n* = 54) versus patients with no grade 3/4 ALT elevation (*n* = 59).

**Figure 3:**
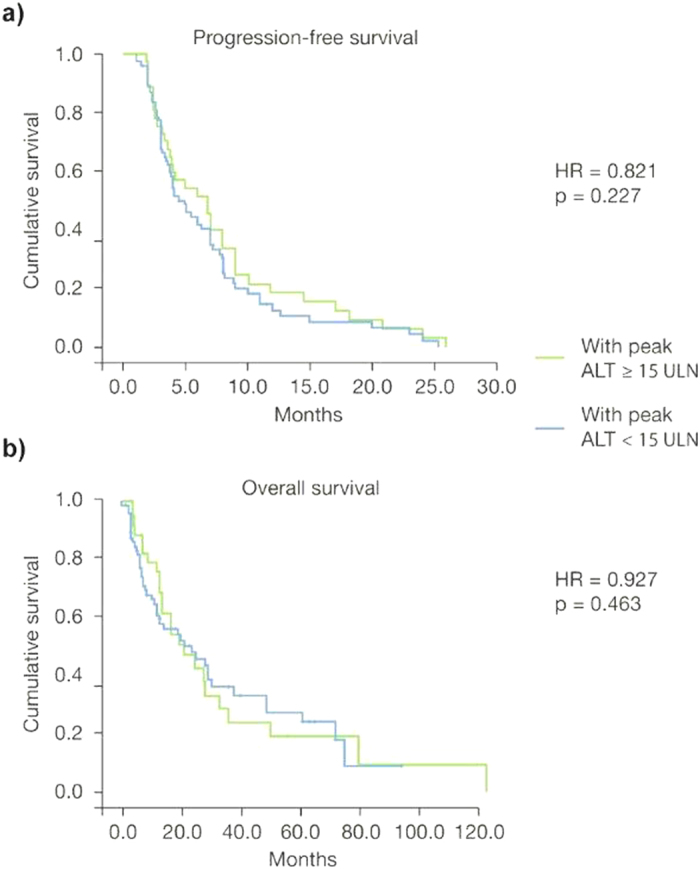
Kaplan-Meier plots of progression-free survival (**a)** and overall survival (**b)** in patients with peak alanine aminotransferase (ALT) elevation ≥15 × upper limit of normal (ULN) (*n* = 33) versus patients with peak alanine aminotransferase (ALT) elevation <15 × ULN (*n* = 80).

Grade 3/4 elevations in ALT/AST during the first two cycles of treatment with trabectedin did not significantly affect treatment outcomes in terms of survival. The hazard ratio (HR) for PFS and OS were not statistically significant [HR = 1.124 (95% CI: 0.761–1.730); *p* = 0.734 and HR = 0.850 (95% CI: 0.513–1.190); *p* = 0.104, respectively] (Fig. 1). Similarly, when the study population was divided into patients with grade 3/4 ALT/AST elevations during treatment versus those with no such abnormalities, the HRs for PFS and OS were also non-statistically significant [HR = 0.791 (95% CI: 0.438–1.390); *p* = 0.309 and HR = 0.930 (95% CI: 0.679–2.007); *p* = 0.811, respectively] (Fig. 2). These findings were confirmed when the HR for PFS and for OS were calculated after dividing the study population into patients with peak ALT/AST elevations ≥15 × ULN versus patients with peak ALT/AST elevations <15 × ULN [HR = 0.821 (95% CI: 0.539–1.965), *p* = 0.227 and HR = 0.927 (95% CI: 0.721–2.310), *p* = 0.463, respectively] (Fig. 3).

## Discussion

**Figure 4:**
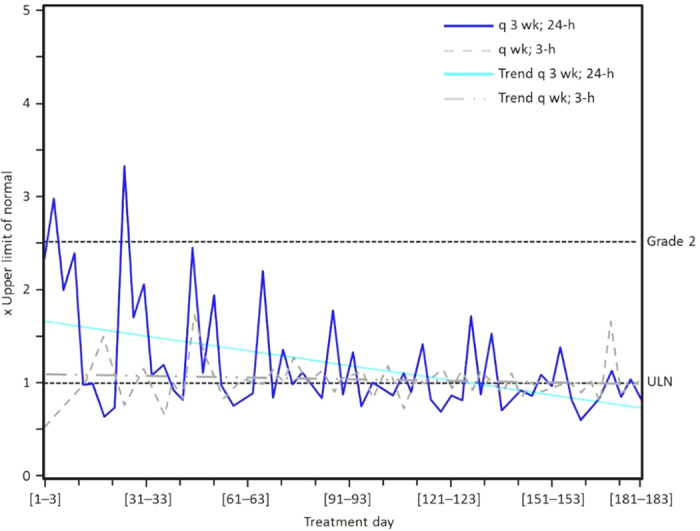
Median alanine aminotransferase (ALT) over treatment cycles in patients with liposarcoma and leiomyosarcoma treated with trabectedin after failing prior anthracycline and ifosfamide treatment. Patients were randomized to trabectedin at a dose of 1.5 mg/m^2^ once every 3 weeks given as a 24-hour intravenous (IV) infusion or 0.58 mg/m^2^ weekly for 3 weeks out of 4 given as a 3-hour IV infusion. ULN = upper limit of normal. Data from Morgan *et al.* 2007^17^.

Trabectedin treatment has shown to be reasonably well tolerated during its phase II and III clinical development. Death due to severe myelosuppression, rhabdomyolysis, acute renal failure, or respiratory failure attributed to treatment occurred infrequently^10^. The most common trabectedin-related AEs reported in the studies are nausea, vomiting, constipation, increases in alkaline phosphatase/AST/ALT, neutropenia, anemia, thrombocytopenia, and fatigue^4^,^5^,^6^,^16^. Among these, grade 3/4 neutropenia and elevation of liver enzymes are reported as the most common toxicities related to dose delay. Although transaminase increases were common with trabectedin, they followed a predictable pattern with a peak elevation at days 5–7 and a return to grade ≤1 at approximately day 15 of each cycle, and with a clear trend towards reduction with subsequent cycles (Fig. 4)^10^,^17^. The analysis per cycle performed in patients treated with the monotherapy regimen in our study showed grade 3 elevations of AST and ALT in 12% and 20% of cycles, respectively. Grade 4 elevations of AST and ALT occurred in 1% and 2% of cycles, respectively. Most transaminase elevations improved to grade 1 or to pre-retreatment levels within 15 days, and less than 2% of cycles had recovering times longer than 25 days. ALT and AST increases did not follow a cumulative pattern but showed a tendency towards less severe elevations over time. In order to manage liver AEs, after the first clinical experience with trabectedin, dexamethasone administration was routinely implemented to improve tolerance. Dexamethasone has been given prior to infusion at the recommended dose of 20 mg 30 min in the majority of clinical studies. In addition, trabectedin dose reductions became mandatory in case of liver toxicity [increase in bilirubin >ULN; and/or alkaline phosphatase (AP) >2.5 × ULN; and/or aminotransferases >2.5 × ULN] between cycles that has not recovered by day 21 of the cycle^4^,^16^. Once a dose has been reduced because of toxicity, dose escalation in the subsequent cycles is not recommended. If any of these toxicities reappear in subsequent cycles in a patient exhibiting clinical benefit, the dose may be further reduced. If more than two dose reductions are necessary, treatment discontinuation should be considered^7^.

In accordance with previous literature, patients of our study reported expected toxicities of treatment with trabectedin. Grade 3/4 increases of ALT were reported in about 40% of patients after the first two cycles of chemotherapy. These liver enzyme elevations did not have a significant impact on PFS (HR = 1.124; *p* = 0.734) and OS (HR = 0.104; *p* = 0.850) of the total study population. This observation was also confirmed in the two sub-analyses that evaluated the same outcomes in patients reporting ALT/AST increases versus those with no increase, and in patients with ALT/AST elevations ≥15 × ULN versus those with ALT/AST elevation <15 × ULN.

These findings are supported by the findings of an exploratory analysis of the safety and efficacy outcomes in pooled data from phase II trials of single-agent trabectedin in heavily and non-heavily pretreated patients with recurrent ovarian cancer. No evidence for a relationship between grade 3/4 increases of ALT/AST during cycles 1 and 2 and efficacy outcomes was found, suggesting that early ALT/AST increase will not negatively affect outcomes^18^.

At present there are no data available in literature or specific trials evaluating the possible correlation between liver tests abnormalities of trabectedin and its efficacy in the treatment of STS, in terms of PFS and OS, which represents a strength of this study. In contrast, the retrospective nature of the study, the reduced number of patients and the heterogeneity of population represent potential limitations of the trial because they may have affected statistical analysis of the results. However, our study findings can provide useful insights into the real-world efficacy, toxicity, and management of patients treated with trabectedin and can represent a good hypothesis to be investigated and validated through further prospective trials.

## Conclusions

Liver tests abnormalities, defined as grade 3/4 transaminase elevations, are common events during treatment with trabectedin. In most cases, AST and ALT elevations are transient and occur within the first two cycles of treatment. According to our current analysis, trabectedin-related liver enzymes elevations do not affect the progression-free or overall survival of patients.

## Additional Information

**How to cite this article**: Vincenzi, B. *et al.* Lack of Correlation Between Liver Tests Abnormalities and Trabectedin Efficacy in the Treatment of Soft Tissue Sarcoma: a Retrospective Study. *Sci. Rep.* **5**, 12077; doi: 10.1038/srep12077 (2015).

## Figures and Tables

**Table 1:** Baseline demographic and clinical characteristics.

Characteristic	*n* = 113
Age, median (range), y	57 (27–79)
Gender, *n* (%)	
Female	42 (37.2)
Male	71 (62.8)
Eastern Cooperative Oncology Group performance status, *n* (%)	
0	43 (38.1)
1	52 (46.0)
2	18 (15.9)
Number of prior chemotherapy regimens	
Median (range)	1 (1–6)
≤2 lines	54 (47.8)
3 lines	45 (39.8)
≥4 lines	14 (12.4)
Tumor histology, *n* (%)	
Liposarcoma	32 (28.3)
Leiomyosarcoma	27 (23.9)
Pleomorphic sarcoma	17 (15.0)
Synovial sarcoma	13 (11.5)
Other	24 (21.2)
Liver metastases at baseline, n(%)	
Yes	27 (20.3)
No	86 (79.7)
